# 1,4-Bis(1*H*-benzimidazol-2-yl)benzene methanol monosolvate

**DOI:** 10.1107/S1600536810049238

**Published:** 2010-12-11

**Authors:** Jiang-Bo Su, Shen Lin, Li-Juan Chen, Ming-Xing Yang, Hua Huang

**Affiliations:** a College of Chemistry and Materials Science, Fujian Normal University, Fuzhou, Fujian 350007, People’s Republic of China

## Abstract

The asymmetric unit of the title compound, C_20_H_14_N_4_·CH_4_O, contains two independent half-mol­ecules, each located on an inversion centre, and a methanol solvent mol­ecule. The benzimidazolyl groups form different dihedral angles [24.0 (1) and 11.6 (1)°] with the plane of the central benzene ring in the two mol­ecules. In the crystal, a two-dimensional network is formed through N—H⋯ N, N—H⋯O and O—H⋯N hydrogen-bonding inter­actions between the benzimidazole units and methanol solvent mol­ecules. π–π stacking inter­actions also occur between the benzimidazole rings of adjacent mol­ecules, with centroid–centroid distances of 3.720 (14) Å and inter­planar distances of 3.53 (1) Å .

## Related literature

For the synthesis of the title compound see: Wu *et al.* (2009 ▶). For the properties and applications of benzimidazoles, see: Tidwell *et al.* (1993 ▶); Salunke *et al.* (1994 ▶); Hoorn *et al.* (1995 ▶); van Berkel *et al.* (1995 ▶); Dinolfo *et al.* (2005 ▶); Yang *et al.* (2008 ▶). For structures of 1,4-bis­(benzimidazol-2-yl)benzene analogues, see: Bei *et al.* (2000 ▶); Wu *et al.* (2009 ▶). For bond lengths and angles in similar structures, see: Matthews *et al.* (1996 ▶); Ozbey *et al.* (1998 ▶).
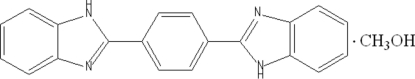


## Experimental

### 

#### Crystal data

C_20_H_14_N_4_·CH_4_O
*M* *_r_* = 342.39
Triclinic, 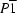

*a* = 7.1730 (14) Å
*b* = 10.599 (2) Å
*c* = 12.260 (3) Å
α = 76.21 (3)°
β = 88.37 (3)°
γ = 77.01 (3)°
*V* = 881.7 (3) Å^3^
*Z* = 2
Mo *K*α radiation
μ = 0.08 mm^−1^
*T* = 293 K
0.31 × 0.16 × 0.12 mm

#### Data collection

Rigaku Mercury CCD diffractometer
Absorption correction: multi-scan (*CrystalClear*; Rigaku, 2002 ▶) *T*_min_ = 0.432, *T*_max_ = 1.000
4565 measured reflections
3139 independent reflections
2577 reflections with *I* > 2σ(*I*)
*R*_int_ = 0.016

#### Refinement

*R*[*F* ^2^ > 2σ(*F* ^2^)] = 0.043
*wR*(*F* ^2^) = 0.119
*S* = 1.04
3139 reflections
240 parameters
H atoms treated by a mixture of independent and constrained refinement
Δρ_max_ = 0.15 e Å^−3^
Δρ_min_ = −0.19 e Å^−3^

### 

Data collection: *CrystalClear* (Rigaku, 2002 ▶); cell refinement: *CrystalClear*; data reduction: *CrystalClear*; program(s) used to solve structure: *SHELXS97* (Sheldrick, 2008 ▶); program(s) used to refine structure: *SHELXL97* (Sheldrick, 2008 ▶); molecular graphics: *SHELXTL/PC* (Sheldrick, 2008 ▶); software used to prepare material for publication: *SHELXL97*.

## Supplementary Material

Crystal structure: contains datablocks global, I. DOI: 10.1107/S1600536810049238/bg2370sup1.cif

Structure factors: contains datablocks I. DOI: 10.1107/S1600536810049238/bg2370Isup2.hkl

Additional supplementary materials:  crystallographic information; 3D view; checkCIF report

## Figures and Tables

**Table 1:** Hydrogen-bond geometry (Å, °)

*D*—H⋯*A*	*D*—H	H⋯*A*	*D*⋯*A*	*D*—H⋯*A*
O1—H1⋯N1	0.93 (3)	1.93 (3)	2.829 (2)	162 (2)
N2—H2*A*⋯N3	0.86	2.03	2.873 (2)	168
N4—H4*A*⋯O1^i^	0.86	1.99	2.855 (2)	179

Symmetry code: (i) 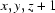.

**Fig. 1.**
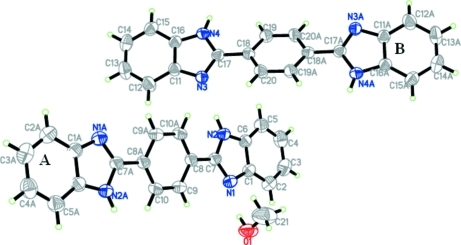
A molecular drawing of (1), showing the atom-numbering scheme. Displacement ellipsoids are drawn at the 50% probability level.

**Fig. 2.**
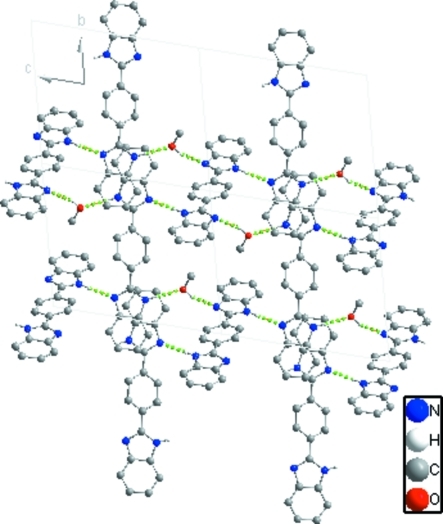
A packing diagram for (1). Broken lines indicate the intermolecular N—H···N hydrogen bonds, N—H···O hydrogen bonds and N—H···O interactions.

## supplementary crystallographic information

### Comment

In earlier communications (Tidwell, *et al.*, 1993; Salunke, *et al.*, 1994; Hoorn, *et al.*, 1995; van Berkel, *et al.*, 1995; Dinolfo *et al.*, 2005; Yang *et al.*, 2008;) it has been reported that the benzimidazole moiety is an important heterocyclic ring not only because of its wide-ranging antivirus activity, its importance in selective ion-exchange resin, but also because of the interest in the coordination chemistry of azoles acting as ligands in transition metal compounds. However, the crystal structure of 1,4-bis(benzimidazol-2-yl)benzene analogues have rarely been reported (Bei, *et al.*, 2000; Wu, *et al.*, 2009;). Herein, we report the crystal structure of the title compound, 1,4-bis(benzimidazol-2-yl)benzene methanol solvate (1).

The structure of title compound is illustrated in Fig. 1. The asymmetric unit contains two different molecules halved by inversion centres at (1/2, 1/2, 1/2) and (0, 1, 0), respectively, and a methanol solvent. Bond lengths and angles have normal values and are comparable to those reported in similar structures (Matthews *et al.*, 1996; Ozbey *et al.*, 1998). The benzimidazoyl moieties form different dihedral angles with the plane of the central benzene ring (24.0 (1)°, 11.6 (1)° for A and B, respectively, Fig. 1). C—N bond lengths in the imidazole ring are in the range 1.328 (2)–1.391 (2) Å, shorter than typical single C—N bond lengths (*ca* 1.48 Å) and longer than typical C=N ones (*ca* 1.28 Å), indicating partial double-bond character. This can be interpreted in terms of conjugation in the heterocycle(Fig. 1, Table 1).

In the solid state the 1,4-bis(Benzimidazol-2-yl)benzene moieties are connected to form a two-dimensional network through intermolecular N—H··· N, N—H···O and O—H···N hydrogen bonds (Fig.2, Table 2). Moreover, there exists π-π stacking interactions between the aromatic and imidazole rings of adjacent molecules, with intercentroid/interplanar distances of about 3.72 (1) Å /3.53 (1) Å, respectively.

### Experimental

All reagents were of AR grade available commercially and used without further purification. To a mixed solvent of polyphosphoric acid (5 ml) and Phosphoric acid (15 ml, 85%) was added benzene-1,4-dicarboxylic acid (1.67 g, 10.0 mmol) and 1,2-diaminobenzene (2.16 g, 20.0 mmol). The mixture was heated slowly to 398 K, and the resulting solution was stirred at 453 K for five hours, and was poured into 300 ml water. Then the mixture was neutralized with 50% sodium hydroxide solution. The crude product was collected by filtration, dried and recrystallized (yield 67%). Crystals suitable for X-ray diffraction analysis were obtained by slow evaporation of a methanol solution.

### Refinement

The (C)H and (N)H atoms of the title compound were placed in calculated positions (C—H = 0.93 and N—H = 0.86 Å) and refined using a riding model, with *U*_iso_(H) = 1.2*U*_eq_(C,*N*). The (C)H atoms of the methanol molecule were placed geometrically (C—H = 0.96 Å) and refined as riding, with *U*_iso_(H) = 1.5*U*_eq_(C). The (O)H atom of the methanol molecule was located in a difference Fourier map and refined with restrained O—H = 0.93 (3) Å and *U*_iso_(H) = 1.5*U*_eq_(O).

### Crystal data

**Table:**

C_20_H_14_N_4_·CH_4_O	*Z* = 2
*M**_r_* = 342.39	*F*(000) = 360
Triclinic, *P*1	*D*_x_ = 1.290 Mg m^−^^3^
Hall symbol: -P 1	Mo *K*α radiation, λ = 0.71073 Å
*a* = 7.1730 (14) Å	Cell parameters from 3188 reflections
*b* = 10.599 (2) Å	θ = 3.0–27.5°
*c* = 12.260 (3) Å	µ = 0.08 mm^−^^1^
α = 76.21 (3)°	*T* = 293 K
β = 88.37 (3)°	Prism, yellow
γ = 77.01 (3)°	0.31 × 0.16 × 0.12 mm
*V* = 881.7 (3) Å^3^	

### Data collection

**Table:**

Rigaku Mercury CCD diffractometer	3139 independent reflections
Radiation source: fine-focus sealed tube	2577 reflections with *I* > 2σ(*I*)
graphite	*R*_int_ = 0.016
ω scans	θ_max_ = 25.2°, θ_min_ = 1.7°
Absorption correction: multi-scan (*CrystalClear*; Rigaku, 2002)	*h* = −8→8
*T*_min_ = 0.432, *T*_max_ = 1.000	*k* = −12→11
4565 measured reflections	*l* = −12→14

### Refinement

**Table:**

Refinement on *F*^2^	Primary atom site location: structure-invariant direct methods
Least-squares matrix: full	Secondary atom site location: difference Fourier map
*R*[*F*^2^ > 2σ(*F*^2^)] = 0.043	Hydrogen site location: inferred from neighbouring sites
*wR*(*F*^2^) = 0.119	H atoms treated by a mixture of independent and constrained refinement
*S* = 1.04	*w* = 1/[σ^2^(*F*_o_^2^) + (0.058*P*)^2^ + 0.2072*P*] where *P* = (*F*_o_^2^ + 2*F*_c_^2^)/3
3139 reflections	(Δ/σ)_max_ = 0.001
240 parameters	Δρ_max_ = 0.15 e Å^−^^3^
0 restraints	Δρ_min_ = −0.19 e Å^−^^3^

### Special details

**Table:**

Geometry. All e.s.d.'s (except the e.s.d. in the dihedral angle between two l.s. planes) are estimated using the full covariance matrix. The cell e.s.d.'s are taken into account individually in the estimation of e.s.d.'s in distances, angles and torsion angles; correlations between e.s.d.'s in cell parameters are only used when they are defined by crystal symmetry. An approximate (isotropic) treatment of cell e.s.d.'s is used for estimating e.s.d.'s involving l.s. planes.
Refinement. Refinement of *F*^2^ against ALL reflections. The weighted *R*-factor *wR* and goodness of fit *S* are based on *F*^2^, conventional *R*-factors *R* are based on *F*, with *F* set to zero for negative *F*^2^. The threshold expression of *F*^2^ > σ(*F*^2^) is used only for calculating *R*-factors(gt) *etc*. and is not relevant to the choice of reflections for refinement. *R*-factors based on *F*^2^ are statistically about twice as large as those based on *F*, and *R*- factors based on ALL data will be even larger.

### Fractional atomic coordinates and isotropic or equivalent isotropic displacement parameters (Å^2^)

**Table:**

	*x*	*y*	*z*	*U*_iso_*/*U*_eq_	
O1	0.4353 (2)	0.72851 (15)	−0.20907 (11)	0.0655 (4)	
H1	0.466 (4)	0.773 (3)	−0.157 (2)	0.099 (9)*	
N1	0.5137 (2)	0.81332 (14)	−0.01667 (11)	0.0462 (4)	
N2	0.4593 (2)	0.81243 (13)	0.16469 (11)	0.0431 (4)	
H2A	0.3999	0.8285	0.2236	0.052*	
N3	0.3135 (2)	0.86666 (13)	0.37352 (11)	0.0404 (3)	
N4	0.34446 (19)	0.84695 (13)	0.55855 (11)	0.0388 (3)	
H4A	0.3731	0.8106	0.6283	0.047*	
C1	0.6775 (3)	0.74325 (16)	0.04753 (14)	0.0438 (4)	
C2	0.8563 (3)	0.67915 (19)	0.01578 (17)	0.0569 (5)	
H2B	0.8818	0.6801	−0.0592	0.068*	
C3	0.9931 (3)	0.6147 (2)	0.09908 (19)	0.0624 (6)	
H3B	1.1126	0.5717	0.0795	0.075*	
C4	0.9576 (3)	0.61209 (19)	0.21223 (18)	0.0584 (5)	
H4B	1.0536	0.5670	0.2660	0.070*	
C5	0.7826 (3)	0.67513 (18)	0.24601 (16)	0.0502 (5)	
H5A	0.7584	0.6733	0.3212	0.060*	
C6	0.6448 (2)	0.74142 (16)	0.16162 (14)	0.0412 (4)	
C7	0.3870 (3)	0.85267 (16)	0.05676 (13)	0.0403 (4)	
C8	0.1892 (2)	0.92809 (15)	0.02858 (13)	0.0394 (4)	
C9	0.1321 (3)	0.98685 (17)	−0.08376 (14)	0.0456 (4)	
H9A	0.2204	0.9784	−0.1401	0.055*	
C10	−0.0540 (3)	1.05739 (17)	−0.11194 (13)	0.0451 (4)	
H10A	−0.0896	1.0955	−0.1870	0.068*	
C11	0.2500 (2)	0.99128 (16)	0.39671 (14)	0.0394 (4)	
C12	0.1739 (3)	1.11565 (18)	0.32434 (16)	0.0541 (5)	
H12A	0.1647	1.1252	0.2472	0.065*	
C13	0.1133 (3)	1.22324 (18)	0.37161 (18)	0.0590 (5)	
H13A	0.0613	1.3065	0.3254	0.071*	
C14	0.1282 (3)	1.21002 (18)	0.48749 (18)	0.0551 (5)	
H14A	0.0844	1.2846	0.5164	0.066*	
C15	0.2059 (3)	1.08975 (17)	0.55992 (16)	0.0484 (4)	
H15A	0.2173	1.0816	0.6368	0.058*	
C16	0.2667 (2)	0.98057 (16)	0.51249 (13)	0.0379 (4)	
C17	0.3672 (2)	0.78377 (15)	0.47268 (13)	0.0358 (4)	
C18	0.4362 (2)	0.63846 (15)	0.48859 (13)	0.0356 (4)	
C19	0.5493 (2)	0.55926 (16)	0.58125 (13)	0.0421 (4)	
H19C	0.5829	0.5982	0.6360	0.063*	
C20	0.3879 (3)	0.57685 (16)	0.40750 (14)	0.0426 (4)	
H20B	0.3125	0.6281	0.3452	0.064*	
C21	0.3492 (5)	0.6260 (3)	−0.1553 (2)	0.0971 (9)	
H21A	0.2685	0.6545	−0.0979	0.117*	
H21B	0.4481	0.5516	−0.1190	0.117*	
H21C	0.2739	0.5965	−0.2043	0.174 (16)*	

### Atomic displacement parameters (Å^2^)

**Table:**

	*U*^11^	*U*^22^	*U*^33^	*U*^12^	*U*^13^	*U*^23^
O1	0.1004 (12)	0.0687 (9)	0.0363 (7)	−0.0341 (8)	0.0012 (7)	−0.0157 (7)
N1	0.0571 (9)	0.0468 (8)	0.0349 (8)	−0.0070 (7)	−0.0014 (7)	−0.0143 (6)
N2	0.0505 (9)	0.0457 (8)	0.0305 (7)	−0.0023 (7)	−0.0035 (6)	−0.0115 (6)
N3	0.0500 (8)	0.0365 (7)	0.0327 (7)	−0.0029 (6)	−0.0045 (6)	−0.0097 (6)
N4	0.0495 (8)	0.0389 (8)	0.0280 (7)	−0.0082 (6)	−0.0005 (6)	−0.0093 (6)
C1	0.0525 (11)	0.0401 (9)	0.0406 (9)	−0.0095 (8)	0.0004 (8)	−0.0138 (7)
C2	0.0608 (13)	0.0566 (12)	0.0539 (12)	−0.0086 (10)	0.0084 (10)	−0.0195 (9)
C3	0.0517 (12)	0.0574 (12)	0.0775 (15)	−0.0048 (10)	0.0052 (11)	−0.0226 (11)
C4	0.0532 (12)	0.0514 (11)	0.0690 (14)	−0.0085 (9)	−0.0142 (10)	−0.0125 (10)
C5	0.0556 (12)	0.0501 (11)	0.0451 (10)	−0.0100 (9)	−0.0089 (8)	−0.0126 (8)
C6	0.0481 (10)	0.0373 (9)	0.0392 (9)	−0.0085 (7)	−0.0028 (7)	−0.0117 (7)
C7	0.0546 (10)	0.0354 (8)	0.0315 (8)	−0.0086 (7)	−0.0031 (7)	−0.0100 (7)
C8	0.0524 (10)	0.0336 (8)	0.0320 (9)	−0.0073 (7)	−0.0060 (7)	−0.0087 (7)
C9	0.0550 (11)	0.0481 (10)	0.0310 (9)	−0.0064 (8)	0.0005 (7)	−0.0090 (7)
C10	0.0585 (11)	0.0451 (10)	0.0281 (8)	−0.0057 (8)	−0.0069 (8)	−0.0063 (7)
C11	0.0417 (9)	0.0366 (9)	0.0400 (9)	−0.0066 (7)	−0.0033 (7)	−0.0108 (7)
C12	0.0682 (13)	0.0429 (10)	0.0471 (11)	−0.0068 (9)	−0.0139 (9)	−0.0063 (8)
C13	0.0660 (13)	0.0350 (10)	0.0711 (14)	−0.0029 (9)	−0.0159 (10)	−0.0092 (9)
C14	0.0553 (12)	0.0420 (10)	0.0718 (14)	−0.0055 (9)	−0.0010 (10)	−0.0254 (9)
C15	0.0542 (11)	0.0471 (10)	0.0491 (10)	−0.0115 (8)	0.0049 (8)	−0.0222 (8)
C16	0.0392 (9)	0.0377 (9)	0.0386 (9)	−0.0096 (7)	0.0021 (7)	−0.0118 (7)
C17	0.0380 (9)	0.0388 (9)	0.0313 (8)	−0.0075 (7)	−0.0006 (6)	−0.0109 (7)
C18	0.0380 (9)	0.0366 (8)	0.0313 (8)	−0.0063 (7)	−0.0011 (7)	−0.0082 (7)
C19	0.0541 (11)	0.0405 (9)	0.0332 (9)	−0.0089 (8)	−0.0084 (7)	−0.0122 (7)
C20	0.0526 (10)	0.0392 (9)	0.0337 (9)	−0.0063 (8)	−0.0122 (7)	−0.0064 (7)
C21	0.141 (3)	0.107 (2)	0.0643 (16)	−0.072 (2)	0.0164 (16)	−0.0224 (15)

### Geometric parameters (Å, °)

**Table:**

O1—C21	1.391 (3)	C9—C10	1.384 (3)
O1—H1	0.93 (3)	C9—H9A	0.9300
N1—C7	1.331 (2)	C10—C8^i^	1.400 (2)
N1—C1	1.391 (2)	C10—H10A	0.9300
N2—C7	1.368 (2)	C11—C16	1.403 (2)
N2—C6	1.378 (2)	C11—C12	1.403 (2)
N2—H2A	0.8600	C12—C13	1.380 (3)
N3—C17	1.328 (2)	C12—H12A	0.9300
N3—C11	1.391 (2)	C13—C14	1.399 (3)
N4—C17	1.3640 (19)	C13—H13A	0.9300
N4—C16	1.385 (2)	C14—C15	1.376 (3)
N4—H4A	0.8600	C14—H14A	0.9300
C1—C2	1.401 (3)	C15—C16	1.395 (2)
C1—C6	1.408 (2)	C15—H15A	0.9300
C2—C3	1.377 (3)	C17—C18	1.475 (2)
C2—H2B	0.9300	C18—C19	1.395 (2)
C3—C4	1.398 (3)	C18—C20	1.402 (2)
C3—H3B	0.9300	C19—C20^ii^	1.386 (2)
C4—C5	1.385 (3)	C19—H19C	0.9300
C4—H4B	0.9300	C20—C19^ii^	1.386 (2)
C5—C6	1.393 (2)	C20—H20B	0.9300
C5—H5A	0.9300	C21—H21A	0.9600
C7—C8	1.469 (2)	C21—H21B	0.9600
C8—C10^i^	1.400 (2)	C21—H21C	0.9600
C8—C9	1.400 (2)		
			
C21—O1—H1	109.8 (16)	C8^i^—C10—H10A	119.7
C7—N1—C1	104.98 (14)	N3—C11—C16	110.02 (14)
C7—N2—C6	107.32 (14)	N3—C11—C12	130.11 (16)
C7—N2—H2A	126.3	C16—C11—C12	119.85 (16)
C6—N2—H2A	126.3	C13—C12—C11	117.72 (18)
C17—N3—C11	104.91 (13)	C13—C12—H12A	121.1
C17—N4—C16	107.26 (13)	C11—C12—H12A	121.1
C17—N4—H4A	126.4	C12—C13—C14	121.53 (18)
C16—N4—H4A	126.4	C12—C13—H13A	119.2
N1—C1—C2	130.63 (17)	C14—C13—H13A	119.2
N1—C1—C6	109.80 (15)	C15—C14—C13	121.84 (17)
C2—C1—C6	119.57 (17)	C15—C14—H14A	119.1
C3—C2—C1	117.88 (19)	C13—C14—H14A	119.1
C3—C2—H2B	121.1	C14—C15—C16	116.80 (17)
C1—C2—H2B	121.1	C14—C15—H15A	121.6
C2—C3—C4	121.92 (19)	C16—C15—H15A	121.6
C2—C3—H3B	119.0	N4—C16—C15	132.62 (16)
C4—C3—H3B	119.0	N4—C16—C11	105.12 (14)
C5—C4—C3	121.46 (19)	C15—C16—C11	122.22 (16)
C5—C4—H4B	119.3	N3—C17—N4	112.68 (14)
C3—C4—H4B	119.3	N3—C17—C18	123.53 (14)
C4—C5—C6	116.64 (18)	N4—C17—C18	123.74 (14)
C4—C5—H5A	121.7	C19—C18—C20	118.34 (15)
C6—C5—H5A	121.7	C19—C18—C17	122.54 (14)
N2—C6—C5	132.09 (16)	C20—C18—C17	119.12 (14)
N2—C6—C1	105.38 (15)	C20^ii^—C19—C18	120.63 (15)
C5—C6—C1	122.52 (17)	C20^ii^—C19—H19C	119.7
N1—C7—N2	112.51 (15)	C18—C19—H19C	119.7
N1—C7—C8	125.12 (15)	C19^ii^—C20—C18	121.03 (15)
N2—C7—C8	122.36 (15)	C19^ii^—C20—H20B	119.5
C10^i^—C8—C9	118.54 (16)	C18—C20—H20B	119.5
C10^i^—C8—C7	121.45 (15)	O1—C21—H21A	109.0
C9—C8—C7	120.00 (16)	O1—C21—H21B	108.1
C10—C9—C8	120.80 (16)	H21A—C21—H21B	107.6
C10—C9—H9A	119.6	O1—C21—H21C	114.6
C8—C9—H9A	119.6	H21A—C21—H21C	108.7
C9—C10—C8^i^	120.66 (15)	H21B—C21—H21C	108.7
C9—C10—H10A	119.7		

Symmetry codes: (i) −*x*, −*y*+2, −*z*; (ii) −*x*+1, −*y*+1, −*z*+1.

### Hydrogen-bond geometry (Å, °)

**Table:**

*D*—H···*A*	*D*—H	H···*A*	*D*···*A*	*D*—H···*A*
O1—H1···N1	0.93 (3)	1.93 (3)	2.829 (2)	162 (2)
N2—H2A···N3	0.86	2.03	2.873 (2)	168.
N4—H4A···O1^iii^	0.86	1.99	2.855 (2)	179.

Symmetry codes: (iii) *x*, *y*, *z*+1.
